# Decoration of β-Cyclodextrin and Tuning Active Layer Chemistry Leading to Nanofiltration Membranes for Desalination and Wastewater Decontamination

**DOI:** 10.3390/membranes13050528

**Published:** 2023-05-19

**Authors:** Umair Baig, Shehzada Muhammad Sajid Jillani, Abdul Waheed

**Affiliations:** Interdisciplinary Research Center for Membranes and Water Security, King Fahd University of Petroleum and Minerals, Dhahran 31261, Saudi Arabia

**Keywords:** membranes, β-Cyclodextrin, terephthaloyl chloride, trimesoyl chloride, interfacial polymerization, desalination, micro-pollutants removal

## Abstract

Given the huge potential of thin film composite (TFC) nanofiltration (NF) membranes for desalination and micro-pollutant removal, two different sets of six NF membranes were synthesized. The molecular structure of the polyamide active layer was tuned by using two different cross-linkers, terephthaloyl chloride (TPC) and trimesoyl chloride (TMC), reacted with tetra-amine solution containing β-Cyclodextrin (BCD). To further tune the structure of the active layers, the time duration of interfacial polymerization (IP) was varied from 1 to 3 min. The membranes were characterized by scanning electron microscopy (SEM), atomic force microscopy (AFM), water contact angle (WCA), attenuated total reflectance Fourier transform infra-red (ATR-FTIR) spectroscopy, elemental mapping and energy dispersive (EDX) analysis. The six fabricated membranes were tested for their ability to reject divalent and monovalent ions followed by rejection of micro-pollutants (pharmaceuticals). Consequently, terephthaloyl chloride turned out to be the most effective crosslinker for the fabrication of membrane active layer with tetra-amine in the presence of β-Cyclodextrin using interfacial polymerization reaction for 1 min. The membrane fabricated using TPC crosslinker (BCD-TA-TPC@PSf) showed higher % rejection for divalent ions (Na_2_SO_4_ = 93%; MgSO_4_ = 92%; MgCl_2_ = 91%; CaCl_2_ = 84%) and micro-pollutants (Caffeine = 88%; Sulfamethoxazole = 90%; Amitriptyline HCl = 92%; Loperamide HCl = 94%) compared to the membrane fabricated using TMC crosslinker (BCD-TA-TMC@PSf). For the BCD-TA-TPC@PSf membrane, the flux was increased from 8 LMH (L/m^2^.h) to 36 LMH as the transmembrane pressure was increased from 5 bar to 25 bar.

## 1. Introduction

Clean water is an enormous challenge for a rapidly growing human population and industrialization [1]. A few technologies have developed over the past years for treating contaminated water resources, which include seawater, domestic and industrial wastewaters. From the various treatment technologies, membrane-based water treatment has shown a huge potential [2,3]. Membrane-based separations have several advantages over conventional treatment methods. The membrane-based separations offer ease of operation and tune-ability, less energy intensive, little footprint and clean output product [4]. Although some of the desalination technologies, such as capacitive deionization (CDI), have proved to be energy efficient, they have certain challenges, such as low electrosorption capacity, slow electrosorption rate and poor cycling stability. Researchers are trying to address such challenges to enhance the chances of transferring CDI to an industrial scale where the potential of Mxenes has been explored [5,6].

Thin film composite (TFC) membranes have been fabricated through interfacial polymerization (IP) on an ultrafiltration (UF) support [7]. IP reaction is generally carried out between an aqueous diamine, such as meta-phenylenediamine (MPD), and a non-aqueous (n-hexane) solution of trimesoyl chloride (TMC) [8,9]. In the case of nanofiltration (NF) membranes, a TFC polyamide membrane is fabricated through IP by using piperazine (PIP) as an aqueous amine crosslinked with TMC [10,11,12]. Since the discovery and success of polyamide TFC membranes in desalination, many studies have been carried out in the literature for improving the performance of the membranes. 

Many strategies, such as tuning the active layers of the polyamide membranes during IP, have been explored in the literature. Many studies have shown that use of different materials, such as MOFs [13], COFs [14], nanomaterials (NMs) [15,16], carbon nanotubes (CNTs) [17], zeolites [18], porous polymers and graphene [19], enhances the performance of the TFC polyamide membranes. In one such effort, Ahmad et. al. used 3,5-diaminobenzoic acid (DABA) during IP in the fabrication of NF polyamide TFC membrane. An NF membrane was fabricated by using PIP and TMC while DABA was added as an additive in the active layer of NF membrane. The inclusion of DABA in the NF membrane resulted in an increase of 20% in water flux during filtration experiments. This increase in permeate flux was attributed to the presence of an additional –COOH group on the DABA which developed a highly hydrophilic membrane [20]. Similarly, UiO-66-NH_2_ was loaded with Ag nanoparticles and the resulting Ag@UiO-66-NH_2_ was incorporated in the polyamide active layer during IP. The resultant Ag@UiO-66-NH_2_ decorated membrane showed excellent antifouling performance with a flux recovery ratio of 95.6%. Moreover, due to the presence of Ag metal, the membrane showed antibacterial rate >95%. Due to the porous nature of Ag@UiO-66-NH_2_, the membrane showed a permeate flux of 47.3 LMH [21]. 

Similarly, many macrocyclic compounds have recently been used during fabrication of NF membranes. The most commonly used macrocycles are crown ethers, cyclenes [22], cyclodextrins (CDs) [23], calixarenes [24], cucurbiturils (CBs) [25] and others. The macrocycles have been explored due to their salient features, such as molecular recognition and multiple functionalities. These macrocycles develop different interactions, such as hydrogen bonding, ionic interactions and hydrophobic interactions. Among these macrocycles, CDs have been used in several separation applications owing to their dual nature having a hydrophobic cavity and an outer hydrophilic surface due to several hydroxyl (–OH) groups on glucopyranose units. Due to these dual features, CDs have been used in different applications, such as water treatment [26], chromatography [27], catalysis [28] and biomedical applications [29]. In a work carried out by Dichtel and co-workers, β-CD (BCD) polymers were used for the removal of organic micro-pollutants, such as pesticides, pharmaceuticals and plastic components [30]. Similarly, BCD polymers were used for the absorption of organic molecules from water [31]. Hence, BCD has huge potential to be explored for the rejection of a variety of pollutants from contaminated feeds. Other macrocycles, such as Cyclene, have been used for the fabrication of polyamide NF membranes for rejecting divalent ions. In one such example, Ming Wang et. Al. developed a NF membrane through IP using Cyclene and TMC on ultrafiltration (UF) PSf support. The fabricated membrane showed salt rejections of 97%, 96.3% and 96.2% for Na_2_SO_4_, MgCl_2_ and MgSO_4_, respectively [32]. 

Another important aspect of NF membranes is tuning the polyamide active layer by altering the chemistry of the monomers used during IP. The pore structure of the NF membranes has been tuned by selecting different combinations of aqueous amines and non-aqueous cross-linkers. The microstructure of the polyamide active layer can be tuned by enhancing the inter-pore connectivity of the polyamide active layer. In a work carried out by Livingston and co-workers, a highly dense polyarylate layer had bisphenol monomers with a rigid structure instead of PIP. The resultant membrane showed higher organic solvent permeate flux and gas permeation [33]. In another work, a copolyamide membrane was developed by adjusting the ratio of bisphenol and PIP, leading to a poly(ester-amide) active layer with enhanced interconnectivity [34]. Similarly, X. Huang et al. developed a polyamide active layer with a hydrolysable side-chain by using PIP derivatives with –COOMe and –COOC_2_H_5_ side chains [35]. The hydrolysis of the side-chain esters led to –COOH groups causing an increase in permeance from 5.7 LMH/ bar to 12.9 LMH/bar. The molecular weight cutoff (MWCO) experiment revealed a decrease in pore size from 358 Da to 270 Da. However, the active layer thickness was reduced from 44.7 nm to 20.7 nm which led to higher permeance. These findings from the literature suggest that altering the chemistry of the active layer during IP is a way forward to the design and development of new membranes with enhanced performance for the rejection of salts and the removal of micro-pollutants, such as pharmaceuticals [36]. Although there have been improvements in membrane performance, the majority of the membranes have been prepared using a set of traditional monomers, such as diamines MPD and PIP, crosslinked with TMC. Therefore, combining the selection of monomers with IP could potentially lead to enhanced membrane performance. The need of the day is to explore a new set of monomers which could open new routes in the era of NF membranes prepared through IP. Therefore, the current work is focused on using unconventional linear aliphatic amines along with incorporation of macrocyclic porous molecules to enhance the NF of the membrane. 

The current study was focused on altering the molecular structure of the polyamide active layer by including BCD, N,N′-bis(3-aminopropyl)ethylenediamine (BAPEDA), terephthaloyl chloride (TPC) and trimesoyl chloride (TMC) in membrane fabrication. An aqueous solution of BAPEDA containing BCD was used during IP. Two sets of different membranes were fabricated by using two different cross-linkers TPC and TMC. Furthermore, the effect of crosslinking time during IP was also studied by varying the time of IP, measuring at 1, 2 and 3 min, leading to two sets of three polyamide TFC NF membranes. Following characterization, the membranes were used for desalination and micro-pollutants (antibiotics) removal.

## 2. Materials and Methods

Beta-cyclodextrin (BCD), terephthaloyl chloride (TPC), Trimesoyl chloride (TMC), polysulfone, triethylamine (TEA) and N,N′-bis(3-aminopropyl)ethylenediamine (BAPEDA) were purchased from Sigma Aldrich, St. Louis, MO, USA. For the filtration test, different salts (MgCl_2_, CaCl_2_, MgSO_4,_ Na_2_SO_4_, NaCl) and pharmaceutically active compounds (Caffeine, Sulfamethoxazole, Amitriptyline, Loperamide) were also bought from Sigma.

The membranes were characterized using an ATR-Fourier-transform infrared spectroscopy (Thermo, Waltham, MA, USA, Smart iTR NICOLET iS10), a scanning electron microscope (JEOL JSM6610LV, Tokyo, Japan), an atomic force microscope (Agilent 550, Amsterdam, The Netherlands) and water contact angle (KRUSS DSA25). The feed and permeate solution were tested using a conductivity meter (Ultrameter II, Hanna, Woonsocket, RI, USA) for salts and a JASCO V-750 UV-Vis spectophotometer for pharmaceutically active compounds. The membranes were tested for their performance using the Sterlitech CF042 Membrane test system, United States of America.

### Membrane Fabrication

**Figure 1:**
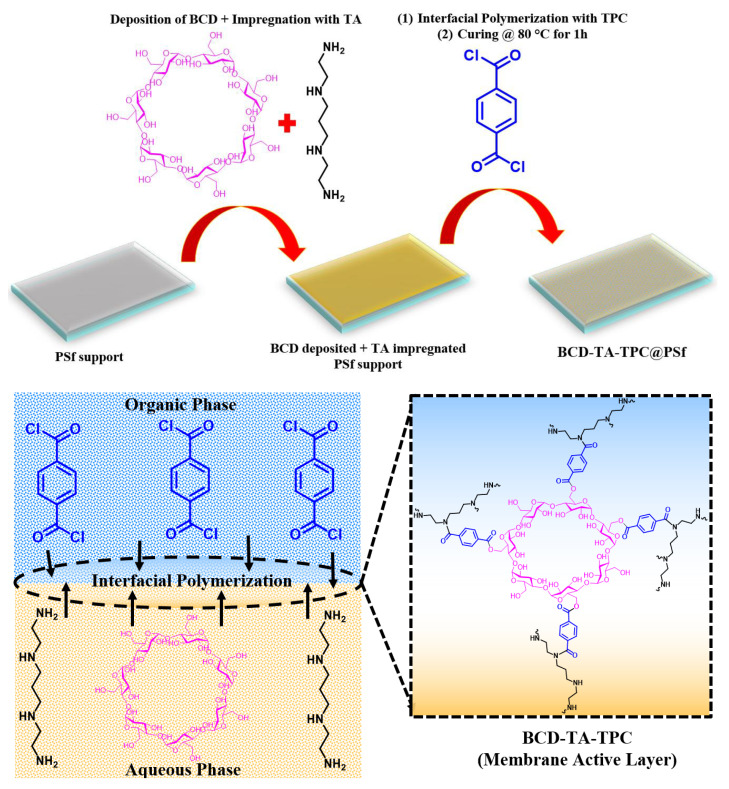
The fabrication phases of TFC NF polyamide membranes.

To evaluate the effect of the crosslinker (TMC vs. TPC), the addition of BCD and the interfacial polymerization time (1, 2, 3 min), six membranes typologies were fabricated. One amine aqueous solution was prepared by keeping the BCD amount constant at 0.1 (*w*/*v*) % and 4 (*w*/*v*) % TEA, and 2 (*w*/*v*) % BAPEDA. The resulting solution was probe sonicated for 15 min to homogenize the contents. Two crosslinker solutions were prepared by dissolving TMC or TPC at a concentration of 0.2 (*w*/*v*) % to n-hexane for interfacial polymerization. Initially, the polysulfone (PSf) layer was cast onto polyester terephthalate (PET) nonwoven fabric via wet phase inversion methodology. Later, it was dipped into the BCD/amine aqueous solution and impregnation was carried out for 10 min using a Cole-Parmer mini rocking shaker. After removing the membrane from the aqueous amine solutions, the rubber roller was used to sweep the extra solution. The membranes were then dipped into the crosslinker either TMC or TPC for 1, 2 or 3 min. This pattern resulted in 6 membranes of different typologies that were denoted as BCD-TA-TMC@PSf-X (X stands for 1, 2 and 3), while the other set was named as BCD-TA-TPC@PSf-X (X stands for 1, 2 and 3). The extra crosslinker solution was washed out by rinsing the membrane with 10 mL of n-hexane. The membranes resulting from the crosslinking of TMC were kept inside an oven at 80 °C for 10 min, where the TPC crosslinked membranes were kept for 1 h at a similar temperature. Before beginning the filtration test, membranes were soaked inside distilled water. Different stages of membrane fabrication are listed in Figure 1.

## 3. Results and Discussion

### 3.1. Membrane Fabrication and Characterization

**Figure 2:**
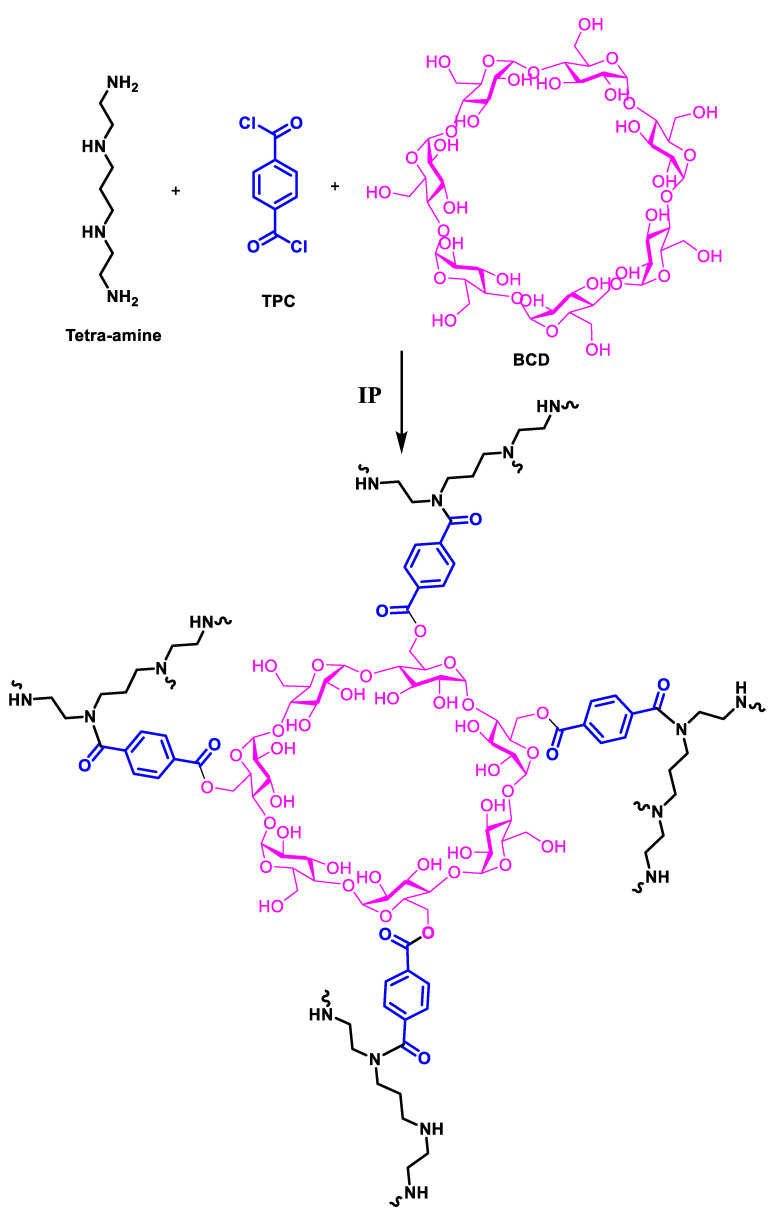
Proposed reaction and structure of the active layer using tetra-amine, TPC and BCD.

The active layer was generated by reacting tetra-amine with TPC and TMC while BCD was added as an additive during IP. The reaction between amine (NH/NH_2_) and acid chloride (–COCl) led to the formation of amide linkage (–CONH), leading to polyamide synthesis. Moreover, hydroxyl groups (–OH) of BCD also reacted with –COCl leading to the covalent linkage of BCD in the active layer of the membrane. The proposed reaction between different reacting monomers is given in the following Figure 2. 

**Figure 3:**
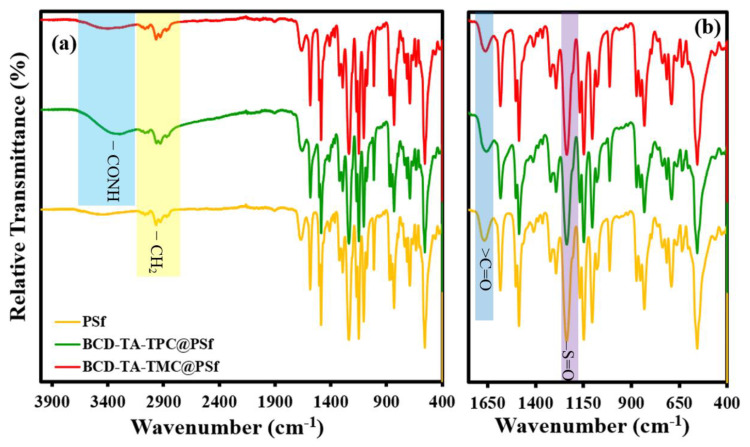
(**a**) ATR-FTIR spectra and (**b**) fingerprint regions of all the fabricated membranes when compared to PSf support.

In order to establish the structure of the best performing membranes (BCD-TA-TPC@PSf and BCD-TA-TMC@PSf), a thorough characterization is extensively described here. The identification of different functional groups present in different fabricated membranes was recorded by an ATR-FTIR spectrum of each membrane, as shown in Figure 3a,b. For the sake of understanding, the ATR-FTIR spectrum of PSf support is also given in Figure 3a,b, where peaks are evident in the aromatic 3000 cm^−1^ and aliphatic 2900 cm^−1^ regions, which are attributed to the benzene rings and –CH_2_ groups present in PSf and PET support. In the case of BCD-TA-TPC@PSf and BCD-TA-TMC@PSf membranes, a new broad peak spanning from 3600 cm^−1^ to 3300 cm^−1^ is evident, which is due to –N-H stretching of newly formed amide (–CONH) linkage in the active layer. Furthermore, the hydroxyl (–OH) groups of the BCD are also overlapped by –N-H stretching of amide linkage. The presence of several other peaks in the fingerprint region is almost like in the case of all membranes. The peak located at around 1650 cm^−1^ to 1680 cm^−1^ can be attributed to carbonyl (>C=O) functional groups of the ester (–COOR) and amide linkages (–CONH). Similarly, the presence of a strong peak at around 1200 cm^−1^ is due to –S=O (sulfone) group of PSf. Hence, the ATR-FTIR spectra of all the membranes confirmed the presence of all the participating functional groups in the structure of the membranes. 

**Figure 4:**
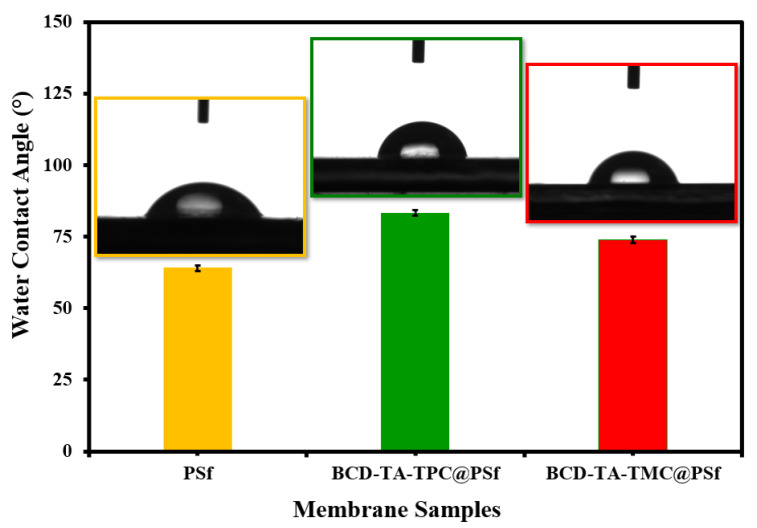
Variations in WCAs of different membranes were prepared during the study.

Surface hydrophilicity of fabricated membranes is highly essential in understanding the filtration performance of the membranes during filtration experiments. For the sake of measuring surface hydrophilicity of the newly developed membrane, WCA of all the membranes along with PSf support was recorded, as given in Figure 4. The WCA of PSf ultrafiltration support was found to be 65° which was increased to 80° in the case of BCD-TA-TPC@PSf, while in in the case of BCD-TA-TMC@PSf, the WCA was found to be 72°. These observations of varying WCAs of membranes revealed that all the membranes were hydrophilic in nature. The variations in WCA can be explained by considering hydrolysis of residual acid chloride (–COCl) groups of cross-linkers. TPC has two acid chloride groups while TMC possesses three acid chloride groups. During IP when the polyamide active layer is growing, the TMC can potentially generate more carboxylic (–COOH) groups compared to TPC. The presence of a higher number of –COOH groups in the case of TMC lowers WCA (72°) in the case of BCD-TA-TMC@PSf compared to BCD-TA-TPC@PSf (80°) (Figure 2).

**Figure 5:**
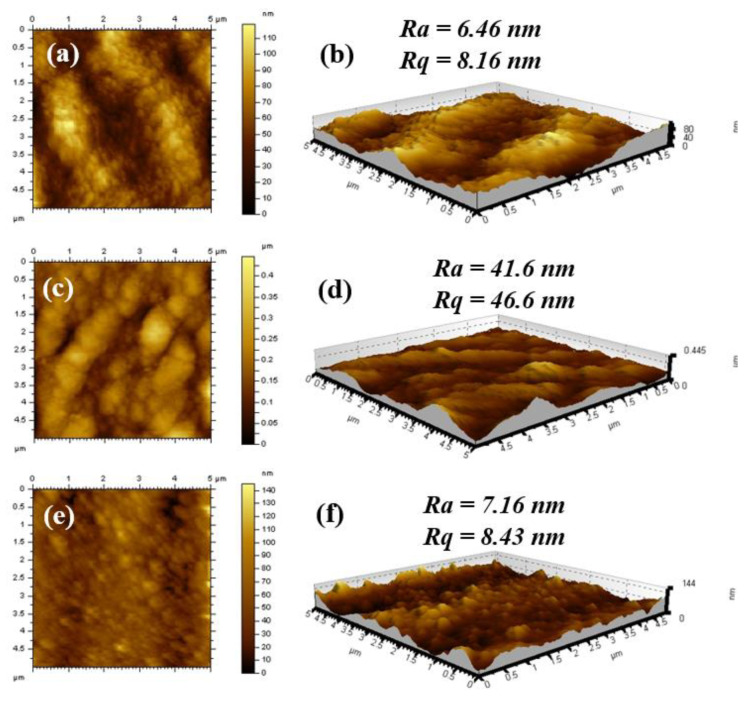
AFM topographical images of (**a**,**b**) PSf support, (**c**,**d**) BCD-TA-TPC@PSf and (**e**,**f**) BCD-TA-TMC@PSf membranes.

Another highly useful surface feature of fabricated membranes is surface roughness as it helps in understanding the performance of the membrane during passage of permeate through the membranes. AFM images of the newly fabricated membranes have been given in Figure 5. The average surface roughness (*R_a_*) and root mean square roughness (*R_q_*) of the membranes are given in Figure 5. The temporal changes in the topography of the PSf support and membranes can be understood by using AFM parameters. In the case of PSf, the values of *R_a_* (6.46 nm) and *R_q_* (8.16 nm) are low compared to BCD-TA-TPC@PSf and BCD-TA-TMC@PSf. The *R_a_* and *R_q_* values of BCD-TA-TPC@PSf were found to be 41.60 nm and 46.6 nm, respectively, while in the case of BCD-TA-TMC@PSf these values were found to be *R_a_* = 7.16 nm and *R_q_* = 8.43 nm. The higher values of *R_a_* and *R_q_* in the case of BCD-TA-TMC@PSf have confirmed that the membrane surface has valleys and ridges providing greater amplitude during roughness measurements. This ridge and valley conformation is ideal for providing appropriate channels during filtration experiments, leading to the rejection of salts and the permeation of pure water. 

**Figure 6:**
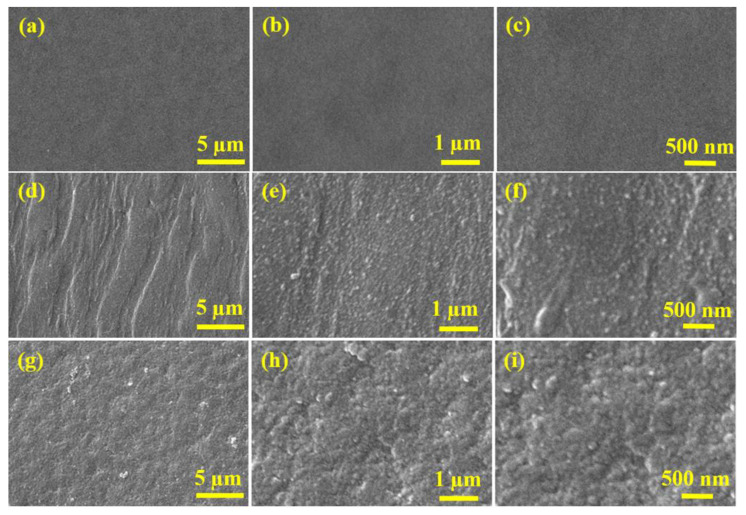
SEM micrographs of (**a**–**c**) PSf support, (**d**–**f**) BCD-TA-TPC@PSf and (**g**–**i**) BCD-TA-TMC@PSf membranes.

To further explore and understand the surface features of the fabricated membranes, the surface morphologies of the membranes were studied through SEM micrographs of the membranes which were recorded at different magnifications, as shown in Figure 6. The surface of the PSf support appeared quite smooth and highly porous in the SEM micrographs (Figure 6a–c). However, the SEM micrographs of BCD-TA-TPC@PSf showed the existence of a continuous polyamide active layer on the PSf support, as the PSf surface morphology is completely masked by the polyamide active layer in the case of BCD-TA-TPC@PSf (Figure 6d–f). Similarly, the BCD-TA-TMC@PSf membrane showed a foamy texture of polyamide active layer (Figure 6g–i). The polyamide active layer was found to be highly dense and beaded in the case of BCD-TA-TPC@PSf compared to BCD-TA-TMC@PSf. These surface morphologies are also augmented by AFM images of the membranes shown in Figure 5. The BCD-TA-TPC@PSf membrane has overall ideal features required for rejecting the salts and permeating clean water as it has a dense polyamide active layer (Figure 6d–f) with ridge and valley confirmation (Figure 5c,d). This is again attributed to extended crosslinking of 4A with TPC, incorporating BCD as additive in the active layer. 

Another salient feature of the membrane surface is the elemental distribution and composition of the membrane. EDX analysis of the PSf support and newly fabricated membranes was carried out, as given in Figure S1. The EDX analysis of PSf (Figure S1a,b) showed the presence of carbon (C), oxygen (O) and sulfur (S). The presence of C is attributed to aromatic rings, while O and S are due to sulfone (O=S=O) groups of PSf. In the case of the BCD-TA-TPC@PSf membrane, in addition to C, O and S, an additional element nitrogen (N) was also found, which confirmed the presence of amide (–CONH) groups in the active layer of the membrane. Similarly, the BCD-TA-TMC@PSf membrane also showed the same elements as found for the BCD-TA-TPC@PSf membrane. Although both the BCD-TA-TPC@PSf (Figure S1c,d) and BCD-TA-TMC@PSf (Figure S1e,f) membranes possessed the similar elemental composition, the amount of N was higher (10.7%) for the BCD-TA-TPC@PSf membrane than for the BCD-TA-TMC@PSf membrane, which had an N percentage of 6.9%. The observation also suggested that there is a dense polyamide active layer grown over the PSf support in the case of the BCD-TA-TPC@PSf membrane. The content of C (73.1%) and O (12.7%) is higher in the case of the BCD-TA-TMC@PSf membrane compared to the BCD-TA-TPC@PSf membrane, with C and O percentages of 70.7% and 12.5%. These variations in the composition of the membranes suggested that the BCD-TA-TMC@PSf membrane has relatively more BCD compared to the BCD-TA-TPC@PSf membrane. 

The distribution of elements in all the membranes detected by EDX analysis is given below in Figure S2. Figure S2a–d demonstrates the elemental mapping results of PSf, where C, O and S are uniformly distributed throughout the entire area of the membrane. However, in the case of the BCD-TA-TPC@PSf (Figure S2e–i) and BCD-TA-TMC@PSf (Figure S2j–n) membranes, an additional element N was also detected, which could be due to the contribution of amines 4A and 4A-3P, and to the growth of a polyamide active layer during IP. 

### 3.2. Nanofiltration Performance of Membranes

The nanofiltration performance of the newly fabricated membranes was studied by using different feeds containing divalent (MgCl_2_, MgSO_4_, CaCl_2_ and Na_2_SO_4_), monovalent (NaCl) salts and micro-pollutants (pharmaceuticals namely Caffeine, Sulfamethoxazole, Amitriptyline HCl and Loperamide HCl) given in Figures S3–S5. The feeds were prepared by dissolving an appropriate amount of salts or micro-pollutants in distilled water. 

**Figure 7:**
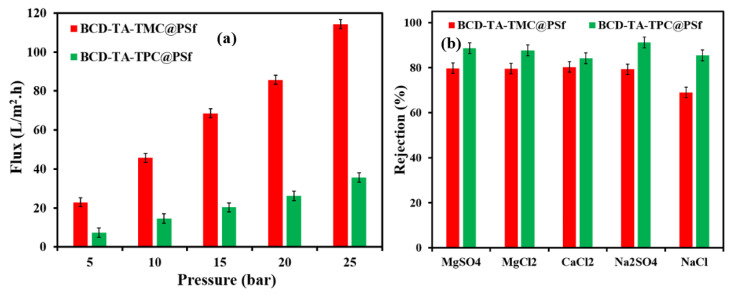
(**a**) Variation in permeate flux as a function of applied feed pressure and (**b**) rejection profiles of the BCD-TA-TMC@PSf and BCD-TA-TPC@PSf membranes.

Prior to the filtration experiments with the above mentioned feeds, the membranes were installed on a crossflow filtration setup and compacted for an hour using distilled water as feed. Initially, the effect of pressure on permeate flux was studied where it was found that the permeate flux increased in a linear manner with increasing transmembrane pressure. The best performing membranes, BCD-TA-TPC@PSf and BCD-TA-TMC@PSf, are described in detail in the upcoming sections. The BCD-TA-TMC@PSf membrane showed higher flux compared to BCD-TA-TPC@PSf (Figure 7a). The value of permeate flux increased from 24 L.m^−2^.h^−1^ (LMH) to 115 LMH when the transmembrane pressure was increased from 5 bar to 25 bar, respectively (Figure 6a). In the case of the BCD-TA-TPC@PSf membrane, the flux was increased from 8 LMH to 36 LMH, with an increase in transmembrane pressure from 5 bar to 25 bar. As anticipated from the flux measurements, the rejection of all salts by the BCD-TA-TPC@PSf membrane was higher compared to the BCD-TA-TMC@PSf membrane. The rejection of Na_2_SO_4_ was found to be the highest (93%) compared to other salts, followed by MgSO_4_ (92%), which was followed by MgCl_2_ (91%), which was followed by CaCl_2_ (84%). The lower rejection of CaCl_2_ can be attributed to the smaller hydration shell (0.334 nm) of Ca^2+^ ions [37] compared to Mg^2+^ and SO_4_^2−^ ions which have hydration radii of 0.86 nm and 0.76 nm, respectively. The rejection of salts depends upon the hydration radii of the permeating ions [38]. Hence, the rejection of MgCl_2_ and Na_2_SO_4_ is higher than CaCl_2_. In the case of monovalent salt, the rejection of NaCl was found to be 85%. The rejection of salts was found to be lower for all the salts in the case of the BCD-TA-TMC@PSf membrane. The rejection and flux performance of membranes suggested that the BCD-TA-TMC@PSf membrane has a relatively loose polyamide active layer with a slightly higher concentration of BCD, as confirmed by EDX analysis. The incorporation of BCD led to higher permeate flux, while a slightly loose polyamide active layer of the BCD-TA-TMC@PSf membrane resulted in lower salt rejection. On the other hand, BCD in the dense polyamide active layer of the BCD-TA-TPC@PSf membrane equip the membrane with reasonable flux and high salt rejection. The increase in permeate flux due to BCD is attributed to outer hydrophilic sphere of hydroxyl groups on the pyranose moieties of BCD.

**Figure 8:**
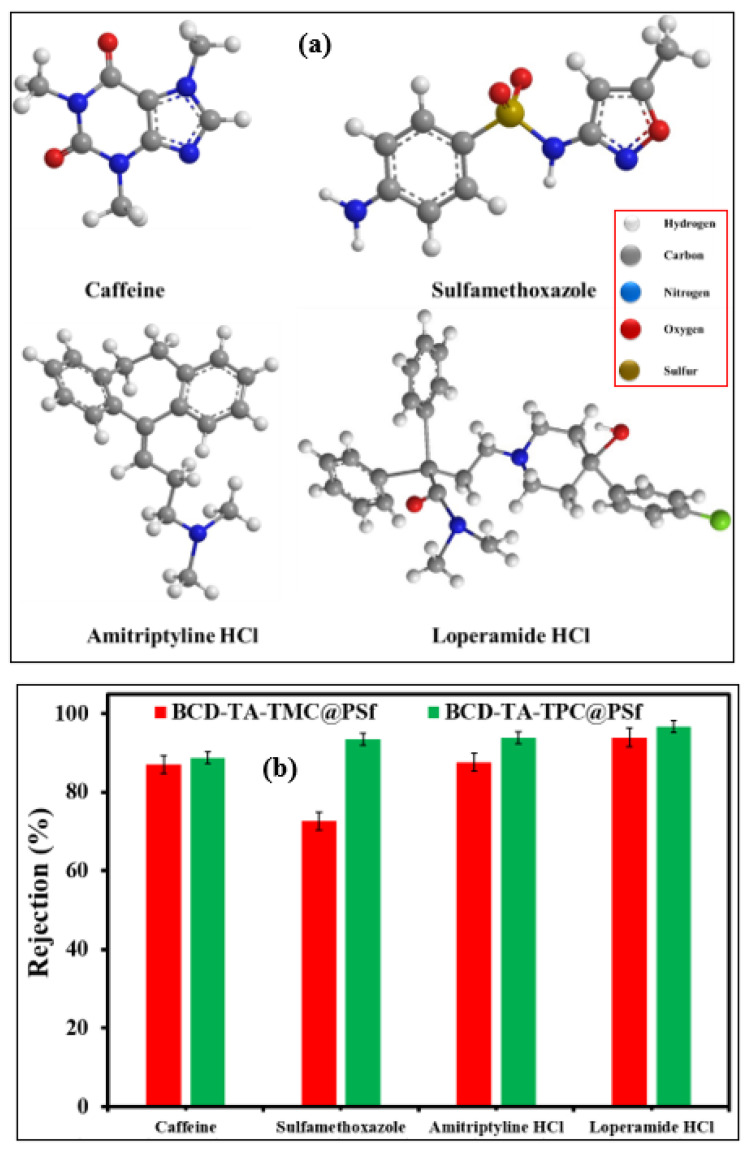
(**a**) Structures of micro-pollutants (pharmaceuticals) with inset showing the legend for atoms in the structure of drugs and (**b**) the rejection profiles of different micros-pollutants by BCD-TA-TMC@PSf and BCD-TA-TPC@PSf membranes.

The concentration of different micro-pollutants, especially pharmaceuticals, is increasing in water bodies at an alarming pace, leading to multidrug resistant bacterial strains. The rejection of the model drugs (Caffeine, Sulfamethoxazole, Amitriptyline HCl and Loperamide HCl) (Figure 7a) have been studied in the current study, as shown in Figure 7b. The rejection of drugs was dependent upon the molecular weight of the drugs. Hence, the rejection of drugs by both fabricated membranes followed a size-exclusion mechanism. In the case of the BCD-TA-TPC@PSf membrane, the Loperamide showed the highest rejection of 94%, while caffeine showed a lower rejection of 88%. The rejection of Sulfamethoxazole and Amitryptiline HCl was found to be 90% and 92%, respectively. Like salt rejection, the BCD-TA-TMC@PSf membrane showed lower rejection for micro-pollutants compared to the BCD-TA-TPC@PSf membrane (Figure 8b). Table 1 shows a comparison of the performances of the BCD-TA-TPC@PSf and BCD-TA-TPC@PSf membranes. 

The performance of the BCD-TA-TPC@PSf membrane was also compared to the performance of membranes in the published literature. The BCD-TA-TPC@PSf membrane has shown either improved or comparable results as shown in Table 2.

## 4. Conclusions

The BCD-TA-TMC@PSf and BCD-TA-TPC@PSf thin film composite nanofiltration membranes were successfully fabricated by interfacial polymerization using two different cross-linkers, terephthaloyl chloride (TPC) and trimesoyl chloride (TMC), reacted with tetra-amine (TA) solution containing β-Cyclodextrin (BCD) for desalination and micro-pollutants removal. ATR-FTIR, SEM, EDS and mapping analysis of the fabricated membranes demonstrated the effective formation of the polyamide active layer on the surface of polysulfone (PSf) support. The thin film composite nanofiltration membranes fabricated using TPC as crosslinker in the presence of TA and BCD showed a better performance for desalination and micro-pollutant removal compared to the thin film composite nanofiltration membranes fabricated using TMC as crosslinker. BCD-TA-TPC@PSf showed rejection of salts, such as Na_2_SO_4_, MgSO_4_, MgCl_2_ and CaCl_2,_ to be 93%, 92%, 91% and 84%, respectively. In the case of BCD-TA-TMC@PSf, the rejection of all of the salts Na_2_SO_4_, MgSO_4_, MgCl_2_ and CaCl_2_ was nearly 80%. Similarly, in the case of pharmaceutical micropollutants in the BCD-TA-TPC@PSf membrane, the higher permeate flux was achieved through the integration of BCD in the polyamide active layer. The BCD-TA-TMC@PSf membrane showed higher permeate flux of 115 LMH compared to the BCD-TA-TPC@PSf membrane, which had a flux of 36 LMH at 25 bar. This might be due to the formation of a slightly loose polyamide active layer with TMC and TA in the presence of BCD. However, due to the formation of a dense polyamide active layer with TPC and TA in the presence of BCD, the BCD-TA-TPC@PSf membrane showed resonable flux and a high level of salts and drug rejections.

## Figures and Tables

**Table 1:** Comparison of different features of the BCD-TA-TMC@PSf and BCD-TA-TPC@PSf membranes.

Feeds	Membranes	
	BCD-TA-TMC@PSf	BCD-TA-TPC@PSf
**Salts**	**Salt Rejections (%)**	**Salt Rejections (%)**
Na_2_SO_4_	80	93
MgSO_4_	80	92
MgCl_2_	80	91
CaCl_2_	80	84
**Pharmaceuticals**	**Pharmaceuticals Rejection (%)**	**Pharmaceuticals Rejection (%)**
Loperamide	92	94
Amitryptiline HCl	85	92
Sulfamethoxazole	70	90
Caffeine	86	88
**Water**	**Permeate Flux (LMH)**	**Permeate Flux (LMH)**
Pure water	115	36

**Table 2:** Performance comparison of the current study with closely published literature.

Sr. No.	Membrane Structure	Salt Rejection	Pharmaceutical Rejection	Ref.
1	Commercial BW30LE-440 and nf90-400	-	85%	[39]
2	Commercial DS5 DK membrane	17–65%	-	[40]
3	3,5-diamino benzoic acid /PA@PS	25.8–60%	80%	[41]
4	PA@Graphene oxide/PS	60–95.2%	-	[42]
5	PA@zeolites/PS	27.7–93.6%	90%	[43]
6	PEES/Ag-NP	31.3–79.4%	-	[44]
7	Diethanolamine/ monoethanolamine/ PA@PS	33.1%	90.8%	[45]
8	BCD-TA-TPC@PSf	93%	94%	This work

## Data Availability

All the data generated related to this work is already presented in the manuscript.

## Supplementary Materials

The following supporting information can be downloaded at: https://www.mdpi.com/article/10.3390/membranes13050528/s1, Figure S1: EDX analysis of (a,b) PSf support, (c,d) BCD-TA-TPC@PSf and (e,f) BCD-TA-TMC@PSf membranes. Figure S2: Elemental mapping analysis of (a–d) PSf support, (e–i) BCD-TA-TPC@PSf and (j–n) BCD-TA-TMC@PSf membranes. Figure S3. Variation of permeate flux of all the membranes with varying applied transmembrane pressure. Figure S4. Rejection different salts by the membranes at 15 bar transmembrane pressure. Figure S5. Rejection different micro-pollutants by the membranes at 15 bar transmembrane pressure.
