# Supplementary material for: Decoration of β-Cyclodextrin and Tuning Active Layer Chemistry Leading to Nanofiltration Membranes for Desalination and Wastewater Decontamination
Source: Membranes (Basel). 2023 May 19;13(5):528. doi: 10.3390/membranes13050528 (PMC10220752; doi:10.3390/membranes13050528)
Supplement: Supplementary file 1 [file membranes-13-00528-s001.zip › membranes-2341749-supplementary.pdf]

membranes

Supplimentary file

Decoration of B-Cyclodextrin and Tuning Active Layer Chemistry Leading to Nanofiltration Membranes for Desalination and Wastewater Decontamination

Umair Baig [1], Shehzada Muhammad Sajid Jillani *,[1] and Abdul Waheed *,[1]

[1]Interdisciplinary Research Center for Membranes and Water Security, King Fahd University of Petroleum and Minerals, Dhahran 31261, Saudi Arabia
* Correspondence: Shehzada.m.jillani@kfupm.edu.sa (S.M.S.J.) abdul.waheed@kfupm.edu.sa (A.W.)

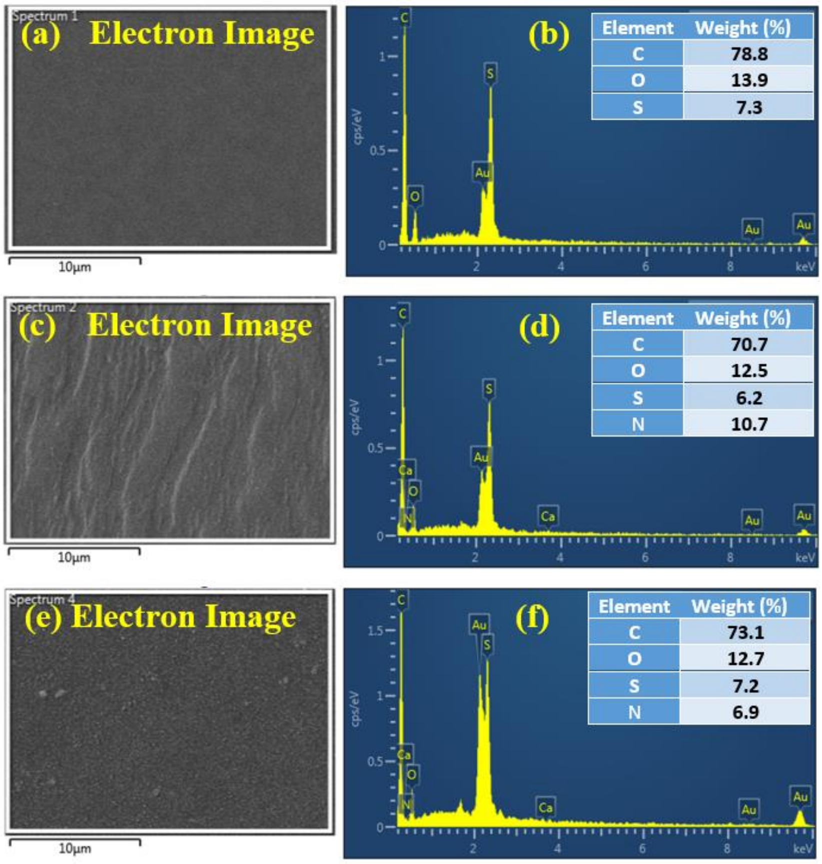

Figure S1. EDX analysis of (a-b) PSf support, (c-d) BCD-TA-TPC@PSf and (e-f) BCD-TA-TMC@PSf membranes.

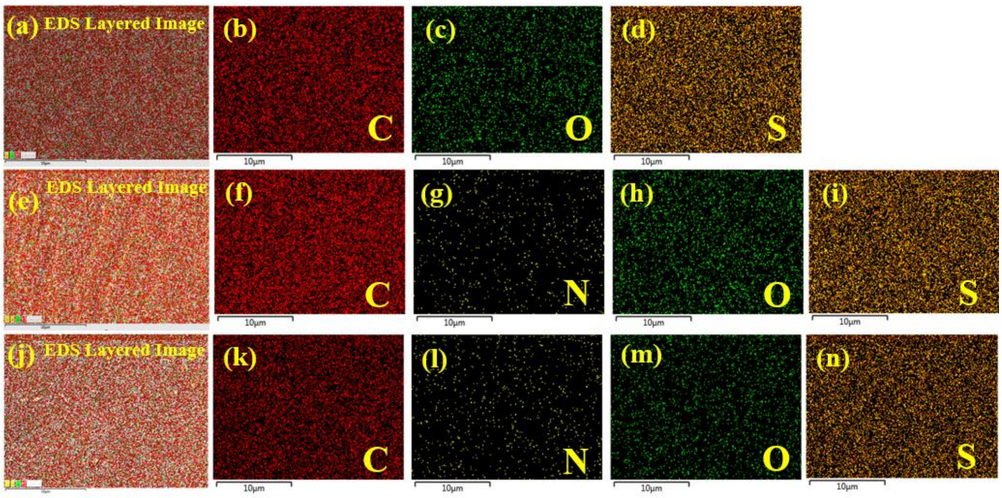

Figure S2. Elemental mapping analysis of (a-d) PSf support, (e-i) BCD-TA-TPC@PSf and (j-n) BCD-TA-TMC@PSf membranes.

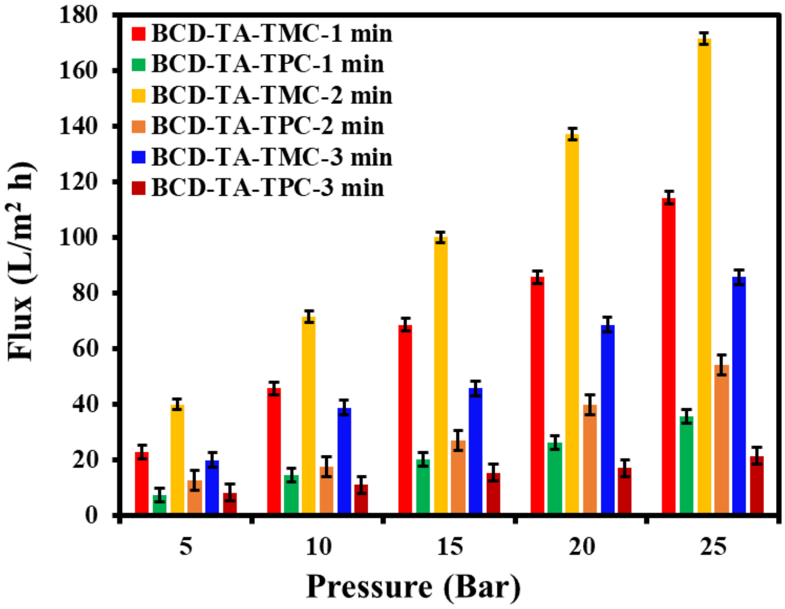

Figure S3. Variation of permeate flux of all the membranes with varying applied transmembrane pressure.

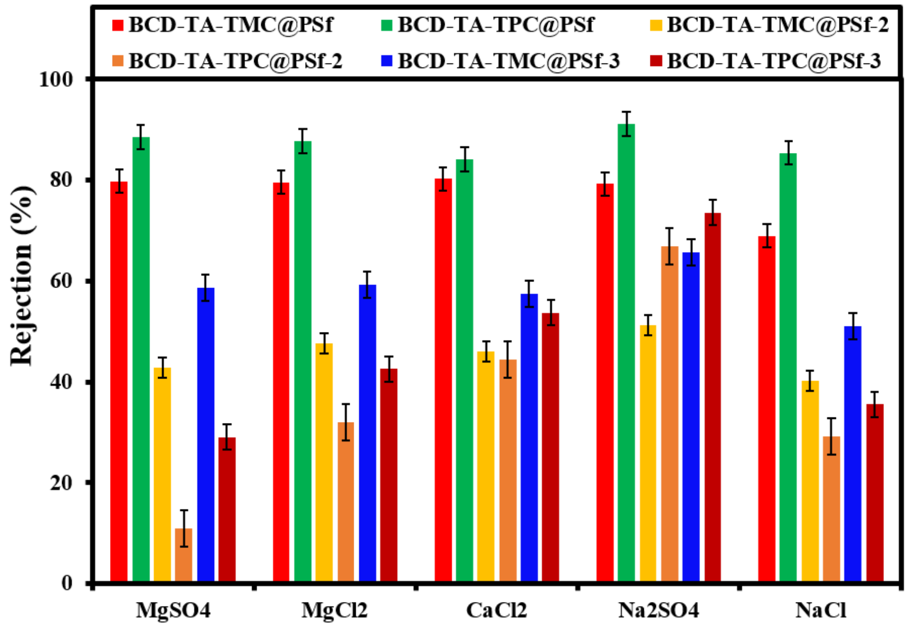

Figure S4. Rejection different salts by the membranes at 15 bar transmembrane pressure.

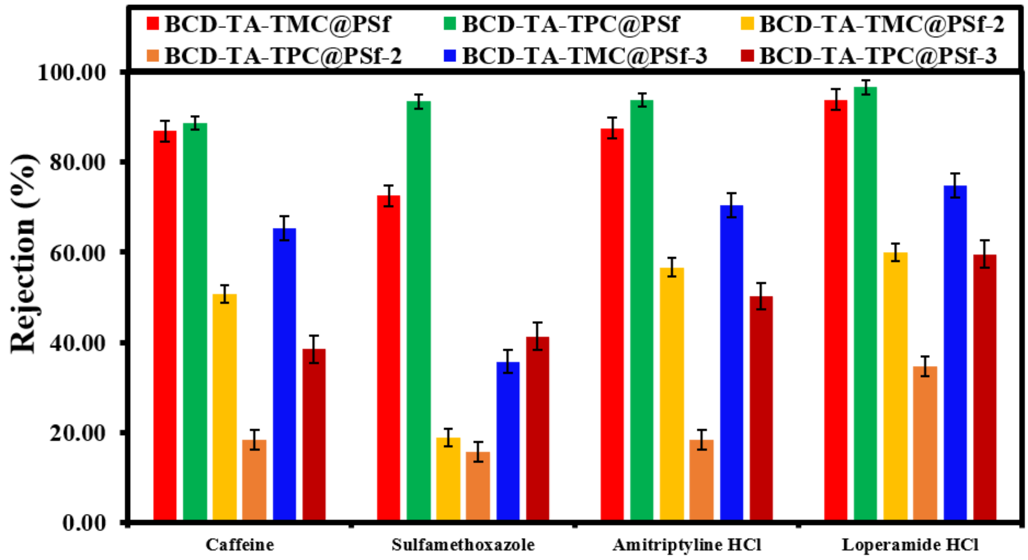

Figure S5. Rejection different micro-pollutants by the membranes at 15 bar transmembrane pressure.
